# Differential proteomics profiling identifies LDPs and biological functions in high-fat diet-induced fatty livers[S]

**DOI:** 10.1194/jlr.M071407

**Published:** 2017-03-29

**Authors:** Mingwei Liu, Rui Ge, Wanlin Liu, Qiongming Liu, Xia Xia, Mi Lai, Lizhu Liang, Chen Li, Lei Song, Bei Zhen, Jun Qin, Chen Ding

**Affiliations:** State Key Laboratory of Proteomics,* Beijing Proteome Research Center, Beijing Institute of Radiation Medicine, National Center for Protein Sciences (PHOENIX Center), Beijing 102206, China; State Key Laboratory of Genetic Engineering,† Collaborative Innovation Center for Genetics and Development, School of Life Sciences, Fudan University, Shanghai 200433, China; Alkek Center for Molecular Discovery,§ Verna and Marrs McLean Department of Biochemistry and Molecular Biology, Department of Molecular and Cellular Biology, Baylor College of Medicine, Houston, TX 77030

**Keywords:** lipid droplet proteins, liver proteomics, isobaric tags for relative and absolute quantitation

## Abstract

Eukaryotic cells store neutral lipids in cytoplasmic lipid droplets (LDs) enclosed in a monolayer of phospholipids and associated proteins [LD proteins (LDPs)]. Growing evidence has demonstrated that LDPs play important roles in the pathogenesis of liver diseases. However, the composition of liver LDPs and the role of their alterations in hepatosteatosis are not well-understood. In this study, we performed liver proteome and LD sub-proteome profiling to identify enriched proteins in LDs as LDPs, and quantified their changes in a high-fat diet (HFD)-induced fatty liver model. Among 5,000 quantified liver proteins, 101 were enriched by greater than 10-fold in the LD sub-proteome and were classified as LDPs. Differential profiling of LDPs in HFD-induced fatty liver provided a list of candidate LDPs for functional investigation. We tested the function of an upregulated LDP, S100a10, in vivo with adenovirus-mediated gene silencing and found, unexpectedly, that knockdown of S100a10 accelerated progression of HFD-induced liver steatosis. The S100A10 interactome revealed a connection between S100A10 and lipid transporting proteins, suggesting that S100A10 regulates the development and formation of LDs by transporting and trafficking. This study identified LD-enriched sub-proteome in homeostatic as well as HFD-induced fatty livers, providing a rich resource for the LDP research field.

Lipid droplets (LDs) are subcellular organelle structures in eukaryotic cells and are composed of a monolayer of phospholipids surrounding a core of neutral lipids, such as triglyceride (TG) and sterol esters (1, 2). All mammalian cells are able to accumulate neutral lipids to form LDs. LDs function in the storage, transport, and metabolism of lipids, supplying essential energy to the organism (3). Growing evidence suggests that LDs are also involved in homeostasis and pathogenesis (4–6). In obesity, the balance between energy intake and expenditure has been broken. While excess energy is mainly stored in LDs of adipose tissue, overaccumulation of LDs in non-adipose tissue, like liver, is associated with fatty liver disease and type 2 diabetes (7). LDs are considered to be organelles, largely based on the findings that they contain a unique proteome that may function in lipid metabolism, LD formation, and differentiation (8). Because few systematic studies on the LD proteome are available (9–12), only a limited number of LD proteins (LDPs) are elucidated for their functions. The best studied LDPs are members of the perilipin (PLIN) family, which is composed of five members from Plin1 to Plin5 (13, 14).

The liver is a central hub for lipid metabolism, accumulating surplus lipids under pathological conditions. Fatty liver is the most common liver pathology, covering the development of hepatosteatosis, progression to nonalcoholic fatty liver disease (NAFLD) and nonalcoholic steatohepatitis, and on to chronic liver diseases, such as cirrhosis, hepatocellular carcinoma (HCC), and liver failure (15). During these processes, hepatocyte LDs undergo dynamic changes; their sizes and numbers presumably reflect the pathological state of the disease (16). The PLIN family has been demonstrated to play important roles in fatty liver disease (7). Plin2-deficient mice displayed reduced hepatosteatosis and lipid levels in NAFLD (17). Similar observations were also made in Plin3 and Plin5 mice (18–20). Fat-specific protein 27 (Fsp27) (21, 22) and hypoxia-inducible protein 2 (Hig2) (23) were also identified as LDPs that may function in hepatosteatosis. Other than NAFLD, hepatosteatosis is a common characteristic in various liver pathological conditions. LD accumulation in liver happens in obesity (ob/ob), diabetes (db/db), fasting, partial hepatectomy, and acute liver injury; however, how the LD sub-proteome varies in these conditions remains ambiguous (24–26).

MS-based proteomics has become a powerful tool for understanding physiology and pathology at the systems level (27). Proteomics allows the dissection of the subcellular organelle proteome, such as the LDPs. Over the last decade, several hundred proteins associated with LDs have been identified by proteomics (9–12, 28–34), which include well-characterized LD-specific proteins, such as the PLIN family, Rabs, and lipid metabolism molecules [e.g., adipose TG lipase (Atgl) and acyl-CoA], as well as ubiquitously expressed proteins, such as actin and tubulin. The identified LDPs from different studies seem to contain common proteins that belong to LDs as well as significantly different proteins whose relationships to LDs are not clear. This may be explained by the dynamic nature of LDs, as active exchange of their components with other subcellular organelles (35) takes place constantly. Thus, LDPs can be categorized into two groups, the “core” LDPs that are highly enriched in LDs and “periphery” LDPs that can be found in other cellular locations, but are enriched in LDs to a lesser extent than the core components. The determination of core and periphery LDPs may shed light on the functional roles of various LDPs. In this study, we employed differential proteomics to identify core and periphery LDPs by comparing the LD sub-proteome with the global proteome. A total of 5,500 proteins were identified in the LD preparation from mouse liver. Using isobaric tags for relative and absolute quantification (iTRAQ) (36), we quantified approximately 5,000 proteins and found that the well-studied Apoc1 and PLIN4 LDPs are highly enriched by more than 100-fold, whereas the other 932 proteins are enriched by more than 2-fold. We carried out a similar analysis for the high-fat diet (HFD)-induced fatty liver and identified core and periphery LDPs for HFD mouse liver, providing a rich resource for the LDP field. We tested the function of an upregulated LDP, S100a10, in HFD liver and found, unexpectedly, that S100a10 knockdown accelerated progression of HFD-induced liver steatosis. Overexpression of S100A10 in vitro revealed a number of transport proteins that are associated with the S100A10 network, revealing a function of S100A10 in lipid transport and trafficking in fatty livers.

## MATERIALS AND METHODS

### Mice

Six-week-old male C57BL/6 mice were purchased from Shanghai Laboratory Animal Center, Chinese Academy of Science (Shanghai, China). HFD (60% fat, 20% protein, and 20% carbohydrate in energy, with a total energy content of 5.24 kcal/g) was purchased from Research Diets Inc. (D12492i; New Brunswick, NJ). The compositions of fat were 316.6 g/kg lard and 32 g/kg soybean oil. Mice were fed with HFD ad libitum for 18 weeks. The establishment of the fatty liver mouse model was performed by following previous reports. (37, 38). All mice were housed in a pathogen-free temperature-controlled micro-environment with 12 h day/night cycles. Mice were maintained with free access to their respective diet until euthanization. All procedures performed were in compliance with the animal care regulations of the State Key Laboratory of Proteomics, Beijing Proteome Research Center, Beijing Institute of Radiation Medicine.

### Cell culture

Oleic acid (OA) was purchased from Sigma. HepG2 cells were cultured in DMEM supplemented with 10% FBS and 1% penicillin-streptomycin (Invitrogen). Transient transfection was performed with Lipofectamine 2000 (Invitrogen) following the manufacturer’s instructions. For in vitro studies, HepG2 cells were cultured for 48 h followed by 1 mmol/l OA (dissolved in BSA) treatment in DMEM.

### Histology

For H&E staining, tissues were fixed in 4% paraformaldehyde overnight, dehydrated, paraffin embedded, and prepared in 5 μm sections. For Oil Red O (ORO) staining, 5 μm frozen sections from snap-frozen liver tissues were fixed in 10% buffered formalin for 3 min. The sections were stained in 0.5% ORO in propylene glycerol and then in hematoxylin for 5 s.

### Confocal imaging

HepG2 cells were plated on coverslips in 12-well plates and transfected with the indicated plasmids. After being treated for 24 h with BSA or OA, coverslips were washed once with PBS and fixed in 4% formaldehyde in PBS for 10 min. Cells were permeabilized for 15 min at room temperature in a staining buffer containing Triton X-100 (0.1%) in PBS and then incubated with DAPI for 10 min. The coverslips were then washed with PBS twice and fixed on slides. Images were captured by ANDOR laser scanning confocal microscope (ANDOR Microscopy Systems).

### Immunoprecipitation-MS

S100A10 was cloned from the human liver cDNA library by standard PCR techniques and subcloned into pcDNA3.1-FLAG. For immunoprecipitation, HepG2 cells were collected 48 h after transfection and lysed in NETN buffer supplemented with 10 mM PMSF (Sigma). The whole cell lysates were immunoprecipitated with 10 μl Protein A beads conjugated with FLAG antibody (Sigma) by incubating for 4 h at 4°C. Immunoprecipitates were washed with lysis buffer and eluted with SDS loading buffer for SDS-PAGE. All samples were prepared by in-gel digestions and analyzed by LC-MS/MS.

### Isolation of LDs from mouse liver

LD isolation was performed according to previous reports. (39, 40) with 2 g of liver that was pooled from three mice (41). Tissues were cut into tiny pieces and washed twice in PBS. The pellet was disrupted in 2.5 ml lysis buffer A [50 mM Tris-HCl (pH 7.4), 1 mM DTT, 250 mM sucrose, 5 mM MgCl_2_, 25 μg/ml spermidine, 1 mM PMSF] using a SPAN bomb (Parr Instrument Company, Moline, IL) for 15 min under 500 psi nitrogen. The lysate was centrifuged for 10 min at 1,000 *g* at 4°C. The supernatant was mixed 1:1 with buffer B (20 mM HEPES, 10 mM KCl, 2 mM MgCl_2_) and centrifuged for 4 h at 154,000 *g* in a Beckman SW41Ti rotor (Beckman Coulter, Inc., Brea, CA) at 4°C. Buoyant fraction (LDs) was washed twice with buffer B and then resuspend in a 2× volume of buffer B.

### Preparation of protein samples and trypsin digestion

Whole tissue proteins were extracted with urea lysis buffer (8 M urea, 100 mM Tris-HCl, and 10 mM PMSF, buffered at pH 8.0) and measured with Bradford assay (Bio-Rad SmartSpec Plus; Bio-Rad Laboratories, Inc., Hercules, CA) according to manufacturer’s instructions. Liver tissues (0.05 g) were lysed in 1 ml 8 M urea on ice for 30 min. Proteins from three mouse livers (300 μg) were dissolved in 300 μl 50 mM ammonium bicarbonate and reduced by adding 3 μl of 1 M DTT for 40 min at 56°C and then alkylated by adding 6 μl of 1 M iodoacetamide for 40 min at room temperature in the dark. Protein samples were digested with trypsin at a mass ratio of 1:50 enzyme/protein overnight at 37°C and the reaction was stopped by the addition of 3 μl of formic acid to a final concentration 1%. LDP was directly extracted from the LD fraction with SDS-PAGE loading buffer. As for 1D-PAGE separation, 20 μg LDP and whole tissue proteins were prepared by in-gel digestion referring to published protocols (42). For iTRAQ labeling, the LDs (75 μg) were subjected to in-solution digestion. After digestion, peptides of LDP were extracted twice in 200 μl of acetonitrile with resuspension in 20 μl of 2% formic acid prior to a second extraction, dried in a Savant SpeedVac, and stored at −20°C until the subsequent MS analysis.

### iTRAQ labeling

Seventy-five micrograms of proteins were subjected to iTRAQ labeling. iTRAQ™ reagent multiplex kit (A4063) was purchased from AB Sciex Pte. Ltd. (Framingham, MA). A 50 μl volume of ethanol was added to each iTRAQ reagent vial (10 ul) and, after vortex mixing, a 30 μl mixture from each iTRAQ vial was transferred to each sample tube. Samples were incubated at room temperature for 2 h and the labeled peptide samples were dried and stored at −20°C until high pH reverse phase (RP) fractionation.

### First dimension high pH RP chromatography

First dimension RP separation was performed on an L-3000 HPLC system (Rigol) using a Durashell RP column (5 μm, 150 Å, 250 × 4.6 mm internal diameter; Agela). Mobile phase A (2% acetonitrile, pH 10.0) and mobile phase B (98% acetonitrile, pH 10.0) were used for RP gradient. Dried peptides were resuspended in 200 μl mobile phase A. The solvent gradient was set as follows: 5–8% B, 2 min; 8–18% B, 11 min; 18–32% B, 9 min; 32–95% B, 1 min; 95% B, 1 min; 95–5% B, 2 min. Tryptic peptides were separated at an eluent flow rate of 1.5 ml/min and monitored at 214 nm. Dried samples were reconstituted in 15 μl of 0.1% (v/v) formic acid and 2% (v/v) acetonitrile in water for subsequent analyses.

### Second dimension low pH RP chromatography coupled with MS/MS measurement

Fractions from the first dimension RP-LC were dissolved in mobile phrase A (0.1% formic acid) and separated on a C18 column (internal diameter, 75 μm). The MS conditions were as follows: For the Triple-TOF 5600, the MS scan range was from *m/z* 350 to 1,250 with a spray voltage of 2,600 V. The top 50 precursor ions were selected in each MS scan for subsequent MS/MS scans with high resolution. MS scans were performed for 0.25 s, and 50 MS/MS scans were performed subsequently for 0.04 s each. The dynamic exclusion for MS/MS was set as 12 s. The CID energy was automatically adjusted by the rolling CID function of Analyst TF 1.5.1. For the Orbitrap Q-Exactive, MS spectra were acquired with a target value of 3E6 and a resolution of 70,000, with a scan range from *m/z* 300 to 1,400. HCD MS/MS spectra were acquired with a resolution of 17,500 and a normalized collision energy of 27%.

### Protein identification

Raw files from Orbitrap Q-Exactive were searched by Proteome Discovery version 1.3 using MASCOT search engine with percolator against the mouse ref-sequence protein database (34,297 proteins, updated on 11-2011). The mass tolerance was set to be 20 ppm for precursor. The tolerance for product ions was set as 20 mmu and 0.5 Da for QE and Velos, respectively. Peptides with at least seven amino acids were retained. Oxidation (Met) and acetyl (N terminus) were chosen as variable modifications; carbamidomethyl (Cys) was chosen as a fixed modification; and one missed cleavage on trypsin was allowed. The target-decoy-based strategy was applied to control peptide level false discovery rates (FDRs) lower than 1% (43). The cutoff ion score for peptide identification was 10. Wiff files from the Triple-TOF 5600 were searched first by ProteinPilot version 4.2 using Paragon search engine against the human ref-sequence protein database (34,297 proteins, updated on 11-2011). Mascot generic format (Mgf) files containing MS peak lists were then exported by ProteinPilot and delivered to Proteome Discovery version 1.3 to search with the same parameters as with QE. For label-free quantification, the protein abundances were estimated by using the intensity-based absolute quantification algorithm (44).

### S100a10 shRNA adenovirus production

The S100a10 shRNA sequences are: shRNA-1, 5′-CCATTGCATGCAATGACTATT-3′; shRNA-2, 5′-CAGAGAAGCTTCTGAGT­TTTA-3′. Before homologous recombination via Gateway system, S100A10-shRNA (NCBI accession number: 6677833) was generated into the plasmid pEntry-EF1a-EGFP-Mir30. The viruses were packaged and propagated in HEK293A cells and then purified by CsCl discontinued density gradient centrifugation. The virus titer was determined using flow cytometry by expression of EGFP in HeLa. Intravenous adenovirus delivery was performed by tail vein high-pressure injections (1 × 10^10^ viral particles per mouse, total volume 1.6 ml in saline) within 5 s. The negative control adenovirus was recombined with shRNA of random sequence. The S100a10 knockdown efficiency was determined by quantitative PCR and multiple reaction monitoring (MRM) MS.

### Quantification for S100a10 in liver tissue

RNA was extracted from 1 g of liver by using Trizol (Invitrogen); reverse transcription of mRNA was performed using a Superscript II kit (Invitrogen) according to the manufacturer’s recommendations. The levels of mRNA were quantified by RT-PCR with SYBR Green PCR Master Mix and CFX96 Touch™ real-time PCR detection system (Bio-Rad Laboratories, Inc.). The relative mRNA levels were determined by the 2^−ΔΔCt^ method and normalized to the housekeeping gene, Actb. Primers used were as follows: S100a10 5′, GCAGGCGACAAAGACCACTTG and 3′, TCTCGGCACTGGT­C­CAGGTCCTTCAT; Actb 5′, AAGCTGTGCTATGTTGCTCTA and 3′, GGATGTCAACGTCACACTTCA. In MRM analysis, three transitions of a precursor (‘PSQMEHAMETMMLTFHR’) were chosen for quantification of protein S100a10 in liver tissues. Collision energy for the precursor was set at 28.6. For each transition, Q1 and Q3 isolation widths of *m/z* 1.0 and 0.7 were employed, respectively.

### Label-free quantification

RP-LC was performed with a home-made column packed with 3 mg C18 (Agela; 3 μm, 150 Å, lot: DC932N2305) in 200 μl pipette tips (Axygen). Thirty micrograms of peptide samples dissolved in 100 μl buffer A were loaded and nine step gradient fractions (6, 9, 12, 15, 18, 21, 25, 30, and 35% ACN in buffer A) were collected. The nine step eluted gradients were mixed into six fractions: (6+25)%, (9+30)%, (15+35)%, 15%, 18%, and 21% and were dried and stored at −20°C until MS analysis on QE. For SDS-PAGE separation, LDPs prepared from three mouse livers (20 μg protein of each group) were separated by 12% SDS-PAGE. Each lane was cut into 12 slices for in-gel digestion (45). Protein quantification was done with a label-free intensity-based absolute quantification approach (44), and total protein normalization was done with a fraction of total (iFOT), which was then multiplied by 10^5^ to obtain iFOT5 for easy visualization.

### Statistics analysis

Data were evaluated using GraphPad Prism (GraphPad Software, La Jolla, CA). Statistical analyses for differences between two experimental conditions were performed with unpaired Student’s (two-tailed) *t*-test; analyses for multiple group comparisons were performed with the one-way ANOVA method. Statistical significance was set at *P* < 0.05.

## RESULTS

### Establishment of a HFD-induced fatty liver model

**Fig. 1.**
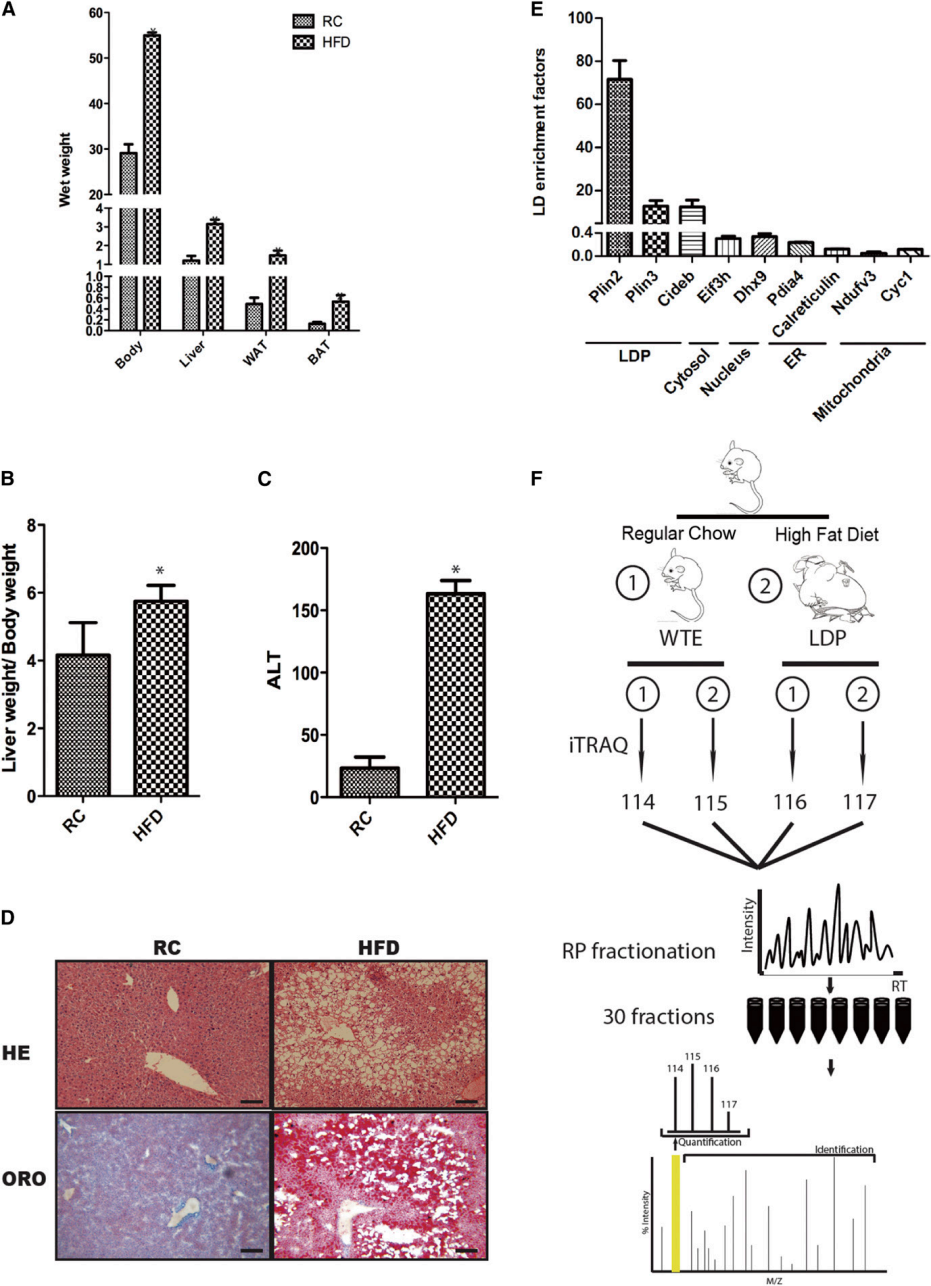
Construction of a fatty liver mouse model induced by HFD. A: Whole body and organ weight with RC and HFD. B: Ratio of liver weight to whole body weight of C57BL/6 mice fed with RC and HFD. C: Serum ALT level. Data are mean ± SD from three mice, n = 5. A two-tailed Student’s *t*-test was performed (*P < 0.05). D: H&E (HE) and ORO staining reveals severe hepatosteatosis and mild liver injury in the mice fed with HFD for 18 weeks (scale bars, 50 μm). E: Enrichment factors of specific proteins to LDs (Plin2, Plin3, and Cideb), nucleus (Dhx9), ER (Pdia4 and calreticulin), mitochondria (Ndufv3 and Cyc1), and cytosol (Eif3h) analyzed by using parallel reaction monitoring. Data are mean ± SD from at least three transitions. F: Streamlined workflow of 4-Plex iTRAQ approach for quantitative profiling of liver global and LD proteome in fatty liver and RC fed mice (114, global control liver; 115, global fatty liver; 116, LD control liver; 117, LD fatty liver).

To establish a fatty liver model, C57BL/6 mice were fed with HFD. After 18 weeks, the whole body and major lipid metabolic organs/tissues, such as liver, white adipose tissue, and brown adipose tissue, were markedly enlarged (**Fig. 1A**). Liver index (liver/body weight ratio) and alanine aminotransferase (ALT) of the HFD group were significantly higher than regular chow (RC)-fed controls (Fig. 1B, C). Liver tissues stained with H&E and ORO showed extensive fat droplet accumulation in the HFD group (Fig. 1D). To evaluate the purity of isolated LDs, we quantified markers that are specific for intracellular organelles, such as mitochondria, endoplasmic reticulum (ER), cytosol, and nucleus in the isolated LDs by using parallel reaction monitoring (28). The level of LD marker, Plin2, was enriched in isolated LDs by over 70-fold, while levels of mitochondria, ER, cytosol, and nucleus markers were significantly reduced compared with whole liver profiling (Fig. 1E, F; supplemental Table S1), indicating a good purity of the LD preparation.

### LDP identification by differential proteomics profiling

**Fig. 2.**
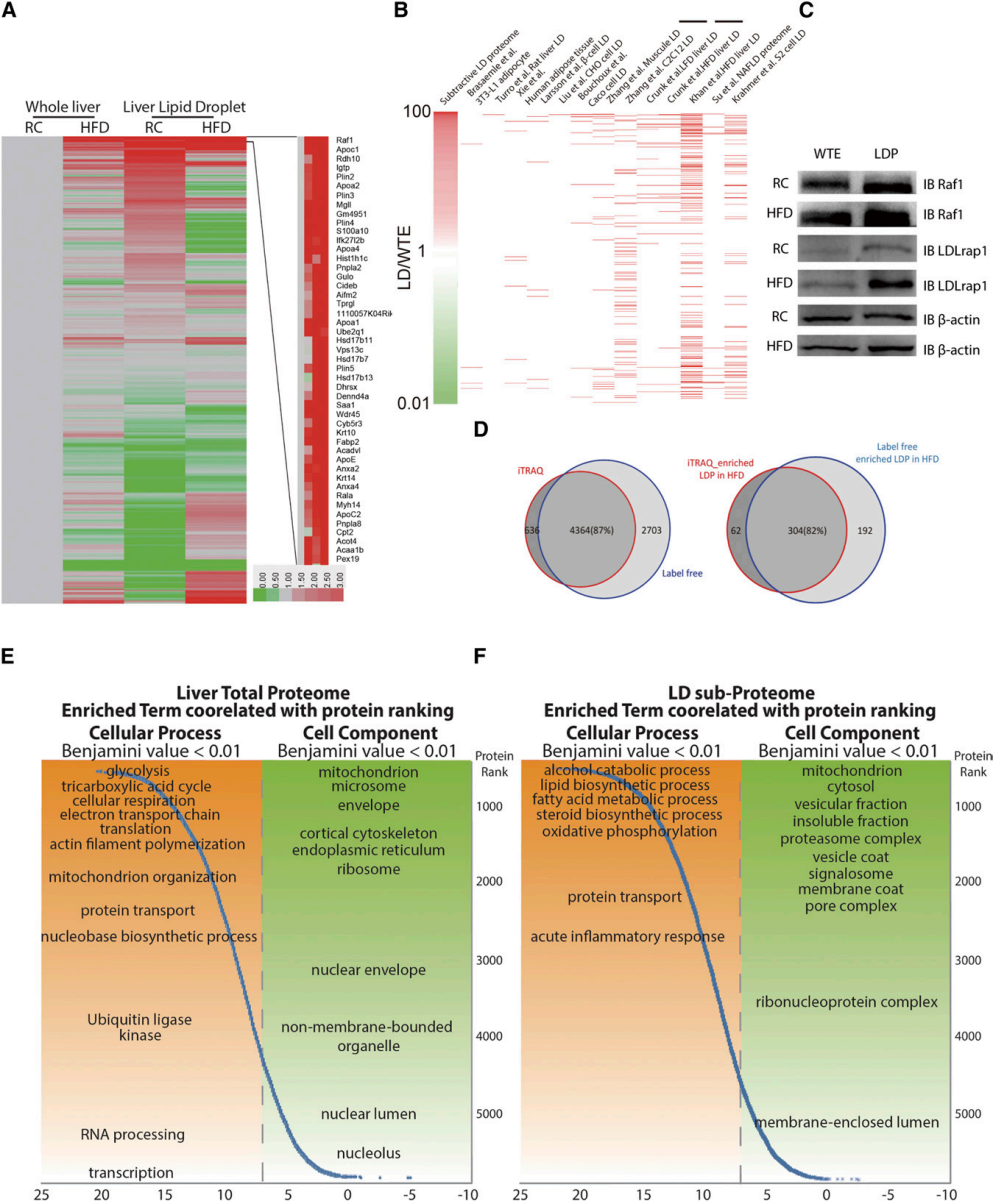
Profiling and quantification of liver global proteome and LD sub-proteome in HFD- and RC-fed mice with iTRAQ. A: An expression heatmap of quantified proteins from global and LD liver proteomes in HFD- and RC-fed mice. The protein expression levels of whole liver extract from RC-fed mice were set as 1 and others were normalized to their corresponding proteins. B: Comparison of LD sub-proteome with published literature. (C) WTE and LDP were extracted from the livers of HFD- and RC-fed mice and the lysates were immunoblotted with anti-Raf1, anti-Ldlrap1, and anti-β-actin antibodies. D: Overlap of proteins identified in liver tissue and LDP between iTRAQ and label-free quantification. E, F: Abundance of detected liver global proteome (E) and LD sub-proteome (F) were quantified and proteins were ranked by abundance. Enrichment of protein GO terms (biological process and cellular component) in each protein abundance quartile was assessed by DAVID software exact test (FDR <0.01 following Benjamini-Hochberg correction). The position of GO terms along the horizontal axis represents enrichment of these terms within the respective protein abundance quartile.

To quantitatively measure LDPs, we extracted proteins from whole livers and the LD fractions of livers, digested them with trypsin, and labeled them with iTRAQ reagents (iTRAQ tag 114, global control liver; 115, global fatty liver; 116, LD control liver; 117, LD fatty liver). We identified 5,519 proteins at 1% FDR at the protein level, and quantified 5,000 of them for their relative abundance (**Fig. 2A**, supplemental Table S2). Protein enrichment factors were determined by comparing their relative abundance in the LD sub-proteome with the liver proteome (supplemental Table S2; iTRAQ tag 116:114). We found that 932 proteins were enriched in LDs by factors of two and above and 101 of them were enriched by greater than 10-fold in LDs. We therefore defined the population with an enrichment factor of >10 as core LDPs. Proteins that were not enriched in the LDP fraction (a total of 1,657 proteins) were likely from contamination during LDP isolation. To determine the reliability of our dataset, we compared our dataset with previous works (supplemental Table S2). Our dataset covered 80.5% of identified LDPs in the HFD mouse liver reported by Khan et al. (12), 93.6 and 93.3% in the low-fat diet and HFD mouse livers, respectively, reported by Crunk et al. (34), and 95.1% of the combined LDP proteomes that were previously identified from different studies. We quantified all identified LDPs using iTRAQ and determined their enrichment in relative abundance compared with the whole liver extract. Our dataset indicated that: *a*) 33 of the 101 core LDPs (supplemental Table S3), including Raf1 and Ldlrap1, have not been reported before (Fig. 2C); and *b*) enrichment factors of previously reported LDPs ranged from 140 to 0.01 (Fig. 2B). By defining LDPs with enrichment factors, this dataset provides a more accurate and expanded list of liver LDPs (supplemental Table S3).

We investigated the reproducibility of LDP identification by performing a biological replicate (with two technique repeats) with label-free quantification. High correlation between technical repeats on both LDP and WTE proteome profiling (supplemental Fig. S1) and high consistency between iTRAQ and label-free biological replicates (Fig. 2D) were obtained. Compared with the iTRAQ dataset, 4,364 out of 5,000 proteins (87%) were identified in the label-free approach (Fig. 2D; supplemental Tables S4-1, S4-2), and 85 of the 101 (85%) core LDPs in RC liver were confirmed by label-free quantifications (supplemental Table S3). We next performed quantitative analyses of global and LD sub-proteome for fatty mouse livers (iTRAQ tag 117:115). Compared with the control group (iTRAQ tag 116:114), LDPs of the HFD-induced fatty livers contained 366 proteins that were upregulated by more than 2-fold, 304 (83%) of which were confirmed as upregulated proteins in the HFD group by label-free quantification (Fig. 2D, supplemental Table S4-3), and 361 proteins were downregulated by more than 2-fold. Consistent with the findings from iTRAQ, label-free quantification also showed that the function of well-studied LDPs and proteins in lipid transport and FA synthesis was highly induced in HFD-induced mouse fatty liver (supplemental Fig. S2A, supplemental Table S5-1). Similarly, label-free quantification showed that enzymes that catalyze the precursor, zymosterol, in vitamin D synthesis were downregulated (supplemental Fig. S2B, supplemental Table S5-2). Comparison of the LD sub-proteome with that of the whole liver proteome revealed several specific over- and under-represented biological processes by LDs (Fig. 2E, F). In terms of cellular components, overrepresented proteins of LDs were associated with the vesicular fraction, membrane coat, and lipoprotein particles (supplemental Fig. S3B, C).

### Differential patterns of global proteome and LD sub-proteome in fatty mouse liver

**Fig. 3.**
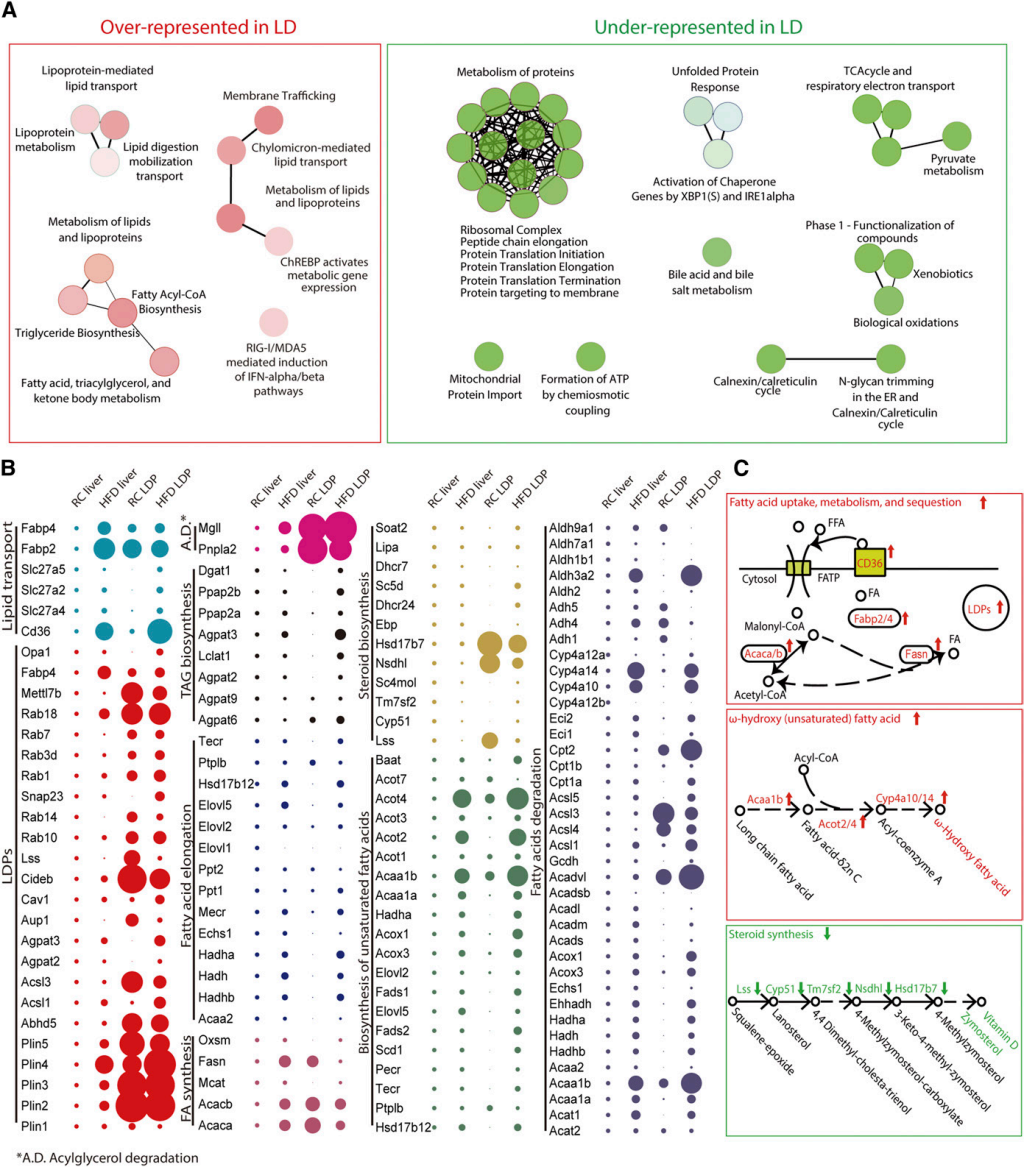
Biological processes mediated by LDPs. A: Over- and under-represented biological processes in mouse liver LD organelles in physiology condition. B: Profiles of lipid metabolism key proteins in the whole liver proteome and LD sub-proteome from both RC and HFD mice. The size of each dot indicates the relative abundance compared with whole liver extract in the RC diet group. Dots are colored according to functional groups. C: Representative models indicating three lipid metabolism pathways that were up- or downregulated in the fatty liver.

Consistent with known functions, LDPs are highly specialized in lipid metabolic processes, such as lipid transport, biosynthesis, and metabolism of phospholipids, sterol, and TG. LDPs are also correlated with glucose metabolism, immune response, and signal transduction (**Fig. 3A**, supplemental Fig. S3A, supplemental Table S6). Of the 200 top LD-enriched proteins, 56 were distributed in the five major groups: membrane trafficking, lipoprotein-mediated lipid transport, metabolism of lipids and lipoproteins, ChREBP-activated metabolic gene expression, and RIG-1/MDA5-mediated induction of the IFN-α/β pathway (supplemental Fig. S4). In comparison, processes that are related to the tricarboxylic acid cycle, protein metabolism, nucleobase metabolism, and transcriptional/translational regulation were significantly underrepresented. Apart from lipid metabolism, proteins involved in stress response to immunity and inflammation were markedly enriched, whereas those involved in the regulation and repairing of ROS, DNA damage, cell death, and basal cellular processes, such as electron transport, cellular respiration, and proliferation, were suppressed in fatty livers (supplemental Figs. S3D, S5), indicating dysregulated cell homeostasis in fatty livers. As illustrated in Fig. 3B and supplemental Fig. S2A, well-studied LDPs and proteins functioning in lipid transport and FA synthesis were markedly induced. In addition, proteins in acyl-glycerol degradation, FA degradation, and unsaturated FA biosynthesis (ω-hydroxy FAs) were upregulated, suggesting an increase in metabolic flow direction from saturated lipid to unsaturated lipid (Fig. 3B). Importantly, five key enzymes in the consecutive enzymatic reactions in steroid biosynthesis, including Lss, Cyp51, Tm7sf2, Nsdhl, and Hsd17b7, were downregulated (Fig. 3B, C), indicating a possible mechanism for vitamin D deficiency in NAFLD.

**Fig. 4.**
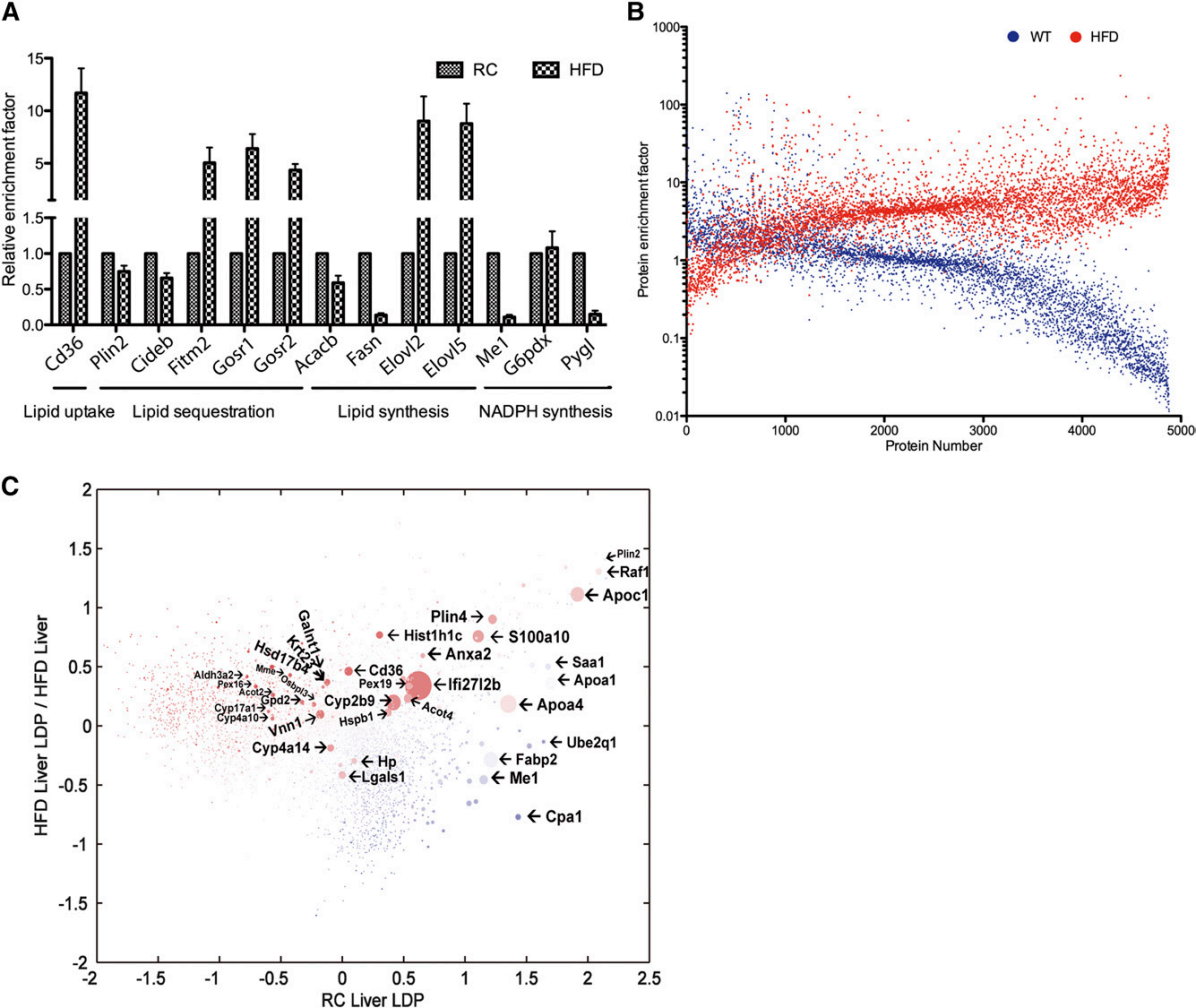
Relative LDP enrichment factors in comparison with representative lipid metabolism proteins (A) and all 5,000 identified proteins in HFD and RC control mice (B). C: Differential proteins positively or negatively regulated in the LD sub-proteome correlated to their global change in the HFD group. LD-enriching preferences are represented by colors. Proteins that are more concentrated in LDs of fatty liver or RC mice are colored red or blue, respectively. Differential ratios of protein abundances in whole liver proteome between fatty liver and RC mice are represented by dot size. Differential proteins whose regulation in the LD sub-proteome positively (red) or negatively (blue) correlated to their global change in fatty liver are marked.

Comparison of the LD sub-proteome between fatty liver and control (iTRAQ tag 117:116) revealed extensive stoichiometry changes in the LD sub-proteome of fatty liver. As the total liver protein ratio in HFD compared with control is 2.6, while the total liver LDP ratio in HFD compared with control is 11.8, this indicated that the LD proteome of the HFD mouse is enhanced by a factor of 4.5 of the total liver proteome. As shown in **Fig. 4A**, the enrichment factor for lipid metabolism “key” proteins in LDs were markedly changed. The exception is Plin2 (the enrichment factor is 0.85), suggesting that the expression of Plin2 is linearly correlated with the LD volume in liver. Surprisingly, global proteome stoichiometry of LDs in RC condition was opposite to that of the HFD pathological condition (Pearson’s *r* = 10^−3^) (Fig. 4B, supplemental Table S7), suggesting that the LD proteome was not as simply enriched and amplified as the LD volume/morphology. LDPs that are positively (red marks) or negatively (blue marks) correlated with their global change in HFD are shown in Fig. 4C, suggesting candidate proteins that may function in hepatosteatosis.

### Knockdown of S100a10 accelerated the hepatosteatosis induced by HFD

**Fig. 5.**
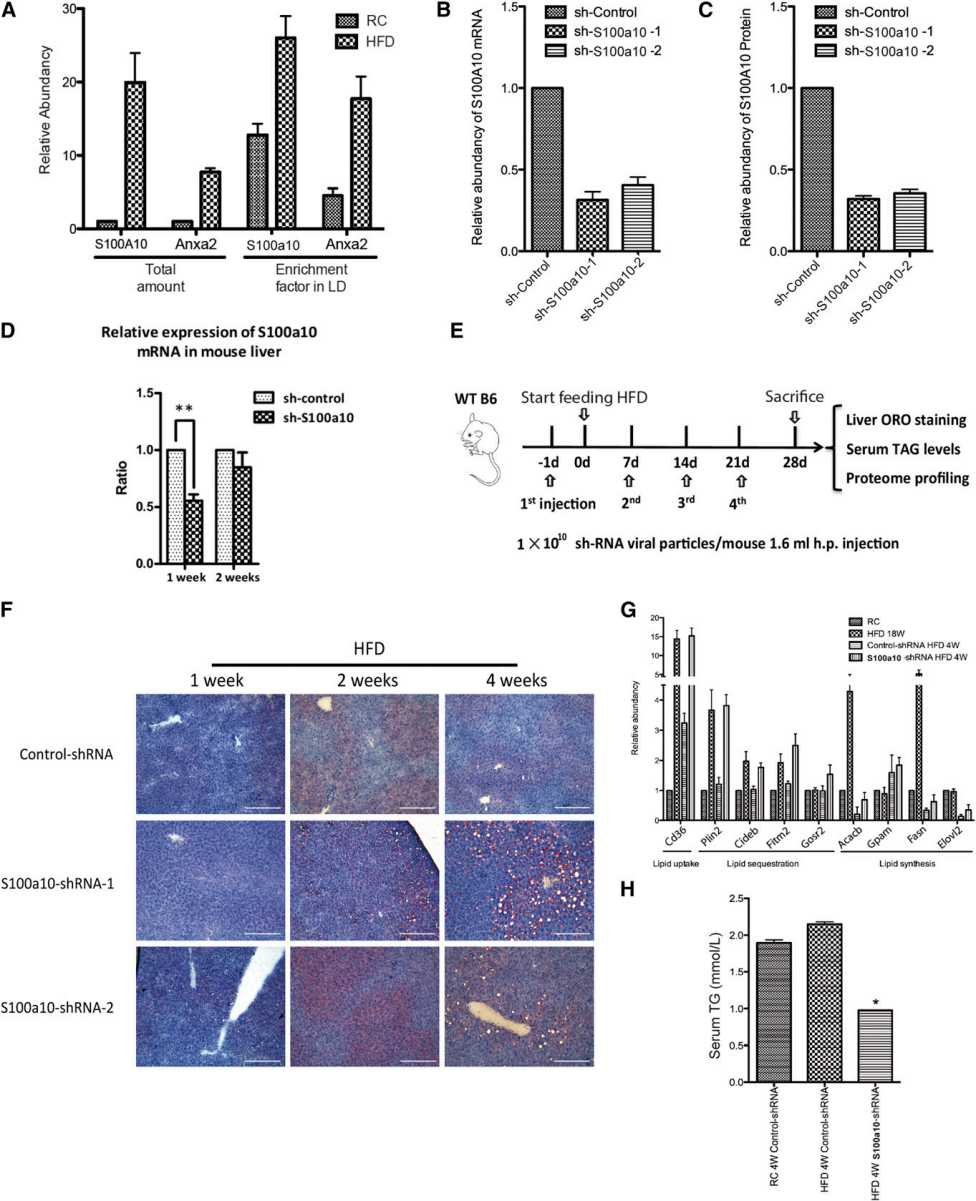
Knockdown of S100a10, a component of annexin A2 heterotetramer complex, accelerated hepatosteatosis induced by HFD. A: Both total abundances and LDP enrichment factors of annexin A2 heterotetramer components were upregulated in the HFD mice. Knockdown efficiency of shRNA-expressing vectors against murine S100a10 was evaluated by Q-PCR (B) and MRM (C). D: Relative expression of S100a10 mRNA in liver tissue treated with adenovirus-mediated shRNA for 1 and 2 weeks. E: Procedure for the injection of recombinant adenovirus shRNA-expression vector in HFD-fed mice. F: ORO staining of liver section in HFD-fed mice administered control-shRNA and S100a10-shRNA (scale bars, 100 μm). G: Expression comparison of key lipid metabolism proteins among RC-fed mice administered control-shRNA for 4 weeks, HFD mice administered S100a10- or control-shRNA for 4 weeks, and wild-type mice fed HFD for 18 weeks. H: Serum TG concentrations from RC-fed mice administered control-shRNA for 4 weeks and HFD-fed mice administered S100a10- or control-shRNA for 4 weeks.

One of the LDPs that is upregulated in fatty liver is S100a10, a membrane protein that forms a hetero-tetrameric complex with annexin A2 (46). The annexin A2/S100a10 complex has been reported as being upregulated in many cancers, including HCC (47). We found that S100a10 and annexin A2 are enriched in LDs by 12.8-fold and 4.5-fold, respectively. Moreover, S100a10 and annexin A2 are also enriched by 19.9-fold and 7.7-fold, respectively, in the liver proteome of the fatty liver, correlating with significant accumulation of annexin A2/S100a10 complex in LD sub-organelles of the fatty liver (Fig. 4C, **Fig. 5A**). The ratio of S100a10 protein between RC and HFD-induced fatty liver was 1:8.9 based on the iTRAQ analysis (supplemental Fig. S6A). Label-free quantification for S100a10 also demonstrated its upregulation in the LDP of HFD-induced fatty liver in mice (supplemental Fig. S6B, supplemental Table S8).

To investigate the role of S100a10 in fatty liver progression, we used adenovirus-mediated shRNA gene silencing in mouse liver to knock down S100a10. Knockdown efficiencies were determined by Q-PCR and MRM for mRNA and protein, respectively (Fig. 5B, C; supplemental Fig. S6C). As shown in Fig. 5D, the shRNA was effective in downregulating S100a10 for 1 week, but not for more than 2 weeks. Interestingly, the levels of serum AST and ALT in mice under shRNA-S100a10 administration were of no significant difference compared with those of the control group (supplemental Fig. S6D). No severe liver injury (supplemental Fig. S6E) and inflammation (48, 49) were observed with H&E staining after 4 weeks of continuous adenovirus injection. Based on these results, we continuously injected S100a10-shRNA recombinant adenovirus weekly for 4 weeks and compared the pathology of hepatosteatosis with that of control shRNA-injected mice (Fig. 5E). As the S100a10 level was elevated in HFD-fed mice, we expected that its loss would result in a slowdown of liver steatosis progression. Surprisingly, the S100a10 knockdown group showed typical hepatosteatosis at 4 weeks after being fed with HFD (Fig. 5F), which was much earlier than the time of 12 weeks, when a similar phenotype was observed in the control shRNA-injected group.

Next, we performed liver proteome analysis of S100A10 knockdown and control knockdown mice after 4 weeks of HFD feeding. Total liver protein extract of RC mice with nonspecific-shRNA, HFD-fed mice administered nonspecific-shRNA, and HFD-fed mice administered S100a10-shRNA were prepared and submitted to proteomics analysis. Over 5,500 proteins were identified and quantified (supplemental Table S9-1). Consistent with the hepatosteatosis phenotype, protein groups involved in lipid metabolism were significantly upregulated in HFD-fed S100a10 knockdown mice, while control knockdown mice showed relatively smaller changes compared to the RC groups (supplemental Fig. S7, supplemental Table S9-2).

Hepatosteatosis could result from excessive activation of lipid uptake, sequestration, and/or synthesis. Cd36, a membrane lipid and FA receptor that facilitates lipid uptake (50), was increased by 4.7-fold in S100a10 knockdown livers, suggesting an increased lipid absorption by S100a10 knockdown. Plin2, a master LDP, as well as Cideb, Fitm1, and G0SR2, which function in lipolysis inhibition and lipid sequestration, were also upregulated in S100a10 knockdown livers (Fig. 5G). To track the lipid source for excessive accumulation of LDs in S100a10 knockdown mouse liver, we measured the serum TG of each group. Serum TG was decreased in the S100a10 knockdown mice compared with RC- and HFD-fed mice administered control-shRNA (Fig. 5H), suggesting that an imbalanced lipid flux from blood to liver in S100a10 knockdown livers may cause the accumulation of hepatic LDs.

### Exogenous expression of S100A10-mediated lipid transport in OA-stimulated HepG2 cells

**Fig. 6.**
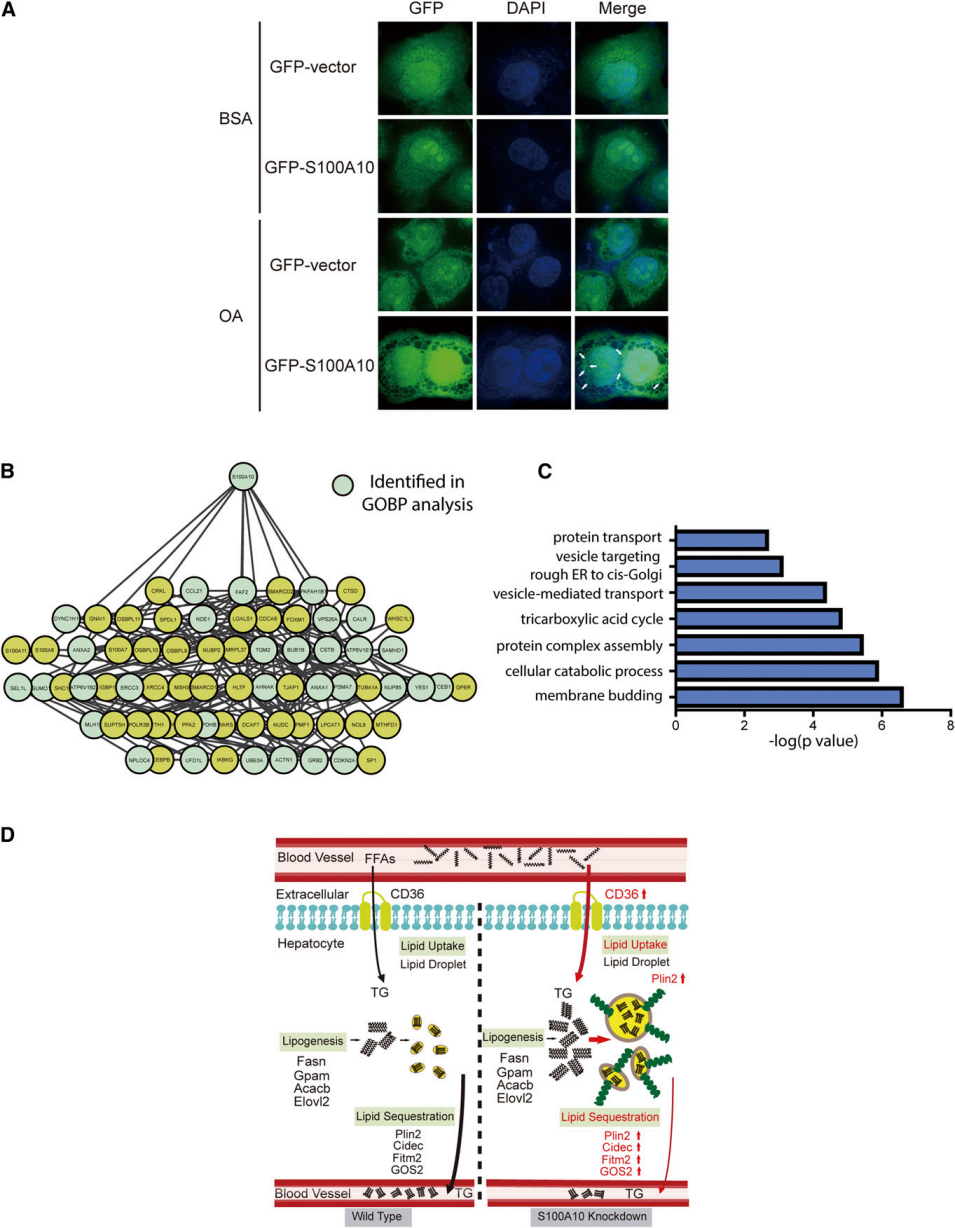
S100A10 promoted lipid transport during OA stimulation in HepG2 cells. A: Control and S100A10 were transfected into HepG2 cells together with enhanced GFP plasmids. After 24 h stimulation, cells were stained with DAPI and imaged by confocal microscopy. The images were captured with a 150× oil objective. B: The interaction network of S100A10 in HepG2 cells. Proteins that are identified in GO/pathway enrichment analysis are colored green, while the nonidentified proteins are colored yellow. C: The GO/pathway enrichment analysis of S100A10 interactome in HepG2 cells. D: Potential mechanism of S100A10 in regulating hepatic lipid metabolism.

To further corroborate the function of S100A10, we conducted overexpression of S100A10 in OA-treated HepG2 cells. We observed that S100A10 accumulated and colocalized with LDs in the cytoplasm of OA-treated HepG2 cells, whereas S100A10 distributed in the cytoplasm in the control cells, suggesting that S100A10 was a reliable candidate of LDPs and involved in LD accumulation (**Fig. 6A**).

We also analyzed the S100A10 interactome in HepG2 cells with immunoprecipitation and LC-MS/MS. A large number of transport proteins, such as DYNC1H1 and NDE1, were identified in the S100A10 immunoprecipitates (Fig. 6B). The GO/pathway analysis of S100A10-induced proteins (fold change over five) indicated that the enriched function terms were membrane budding, cellular catabolic process, tricarboxylic acid cycle, and vesicle-mediated transport (Fig. 6C, supplemental Table S10), suggesting that S100A10 is a functional mediator for lipid metabolic process and contributes to lipid transport and trafficking. In conclusion, our findings implicated a functional role of S100A10 in lipid metabolism and suggested a potential mechanism for S100A10 in lipid sequestration and transport (Fig. 6D).

## DISCUSSION

LDs universally exist in organisms and accumulate, especially in the metabolism organs/tissues. Comprehensive proteome studies have focused mainly on lower invertebrates, such as in *Drosophila* (9, 28). An in-depth LD proteome dataset in higher vertebrates was not readily available. Only a limited number of LDPs were reported (12, 29–31, 34). The most comprehensive list of LDPs was reported by Khan et al. (12), which includes 1,520 LDPs in mouse livers following high-fat feeding using iTRAQ. Additionally, 1,070 and 1,481 LDPs were identified in the C2C12 myoblasts (29) and *Drosophila* S2 cells (28), respectively. Our study differs from the previous ones in that we defined LDPs as proteins that were significantly enriched in abundance from whole proteome, as opposed to those identified from isolated LD fraction (10, 11, 29–34). The fact that our dataset covered over 80% of all previously identified LDPs suggests that our LD sub-proteome is reliable. We further classified 101 proteins as core LDPs and another 823 proteins as periphery LDPs based on different enrichment factors, taking into consideration the dynamic nature of the LD organelle.

Hepatosteatosis is the first step in the pathophysiologic continuum of a number of liver diseases, such as NAFLD (51), alcoholic liver disease (52), and viral hepatitis (53), among others, suggesting the vulnerability of liver lipid metabolism in response to exogenous stimuli. Our data indicated that metabolic pathways in fatty liver were highly induced in TG metabolism, while they were suppressed in phospholipid metabolism. This was consistent with a lipidomics report (54) and suggested a metabolic imbalance between neutral lipid and phospholipid in fatty liver.

The association between vitamin D deficiency and NAFLD has been increasingly recognized (55). Vitamin D level is low in NAFLD patients (56, 57) and administration of vitamin D is efficacious in preventing NAFLD (58). Our data showed that five key enzymes involved in generating vitamin D precursors are decreased in fatty liver, which indicated a deficiency in precursor zymosterol in vitamin D synthesis, reenforcing the relationship between vitamin D and NAFLD reported before (56). This observation suggested that the decrease of vitamin D in fatty liver might be irreversible, because a chain of key metabolic enzymes was downregulated. Moreover, our findings indicated that pathways of immune and inflammatory response were stimulated, while regulation and reparability of ROS, DNA damage, cell death were suppressed in the fatty mouse liver.

With a catalog of LDPs in fatty liver, we focused on one candidate, S100a10, for further functional characterization. Both S100a10 and its binding partner, annexin A2, were enriched in liver LDs in physiological conditions, and were further increased with HFD. Based on these results, we initially hypothesized that knockdown of S100a10 might alleviate hepatosteatosis induced by HFD feeding. Opposite to our predictions, hepatosteatosis was accelerated in HFD when S100a10 was knocked down, suggesting that S100a10 may inhibit hepatosteatosis. We speculated that S100a10 played a compensatory role in the process, that its upregulation was a consequence rather than the cause of hepatosteatosis. Further proteomics analyses seemed to suggest that excessive lipid uptake and lipid sequestration, but not lipid synthesis, were contributing factors for the accelerated hepatosteatosis in HFD-fed mice lacking hepatic S100a10. These analyses were consistent with reduced serum TG, implying an imbalanced lipid flux from blood to liver. These postulations were further corroborated with our in vitro findings that S100A10 colocalized with LDs and coexpressed with a number of transport proteins, demonstrating that S100A10 was a LDP that potentially mediated lipid transport and trafficking. Moreover, S100A10 was found to be unregulated in HCC patients (47) and positively correlated with tumor progression, suggesting a close relationship between S100A10 and liver disease. Recently, Svenningsson et al. (59) showed that S100A10 interacts with SMARCA3 and forms a transcriptional machine in regulating gene expression. The detailed molecular mechanism by which S100A10 regulates LDPs and lipid metabolism proteins deserves further investigation.

## Supplementary Material

Supplemental Data
