# Supplementary material for: Differential proteomics profiling identifies LDPs and biological functions in high-fat diet-induced fatty livers
Source: J Lipid Res. 2017 Mar 29;58(4):681–94. doi: 10.1194/jlr.M071407 (PMC5392744; doi:10.1194/jlr.M071407)
Supplement: Supplemental Data [file supp_58_4_681__index.html]

Differential proteomics profiling identifies lipid droplet proteins and biological functions in high fat diet induced fatty livers — Differential proteomics profiling identifies LDPs and biological functions in high-fat diet-induced fatty livers — Supplemental Data 

# Differential proteomics profiling identifies LDPs and biological functions in high-fat diet-induced fatty livers

## Supplemental Data

- Supplemental Table S1 (.xlsx, 126 KB) - Evaluation the purity of isolated LD in intracellular organelles by using parallel reaction monitoring (PRM).
- Supplemental Table S1 (.xlsx, 536 KB) - Proteins identified and quantified in whole liver protein and LDP both in regular chaw (RC) and fatty livers.
- Supplemental Table S3 (.xlsx, 20 KB) - The enrichment factors of 101 ???core??? LDPs in RC group.
- Supplemental Table S4 (.xlsx, 3.7 MB) - Proteins identified in both iTRAQ and label-free approach.
- Supplemental Table S5 (.xlsx, 16 KB) - Relative abundance of proteins in LDP and other 6 lipid metabolic processes.
- Supplemental Table S6 (.xlsx, 32 KB) - Over- and under-represented biological processes of LDPs.
- Supplemental Table S7 (.xlsx, 516 KB) - Enrichment factors of proteins identified in LDP of RC and HFD groups.
- Supplemental Table S8 (.xlsx, 12 KB) - Label free quantification for S100a10 in LDP of RC and HFD groups.
- Supplemental Table S9 (.xlsx, 371 KB) - Proteomic analysis of proteins identified in S100a10 knockdown and control mice liver after 4 weeks of HFD feeding.
- Supplemental Table S10 (.xlsx, 12 KB) - Biological processes analysis of S100A10 interactome
- Supplemental figures, figure legends and table legends (.pdf, 1.1 MB) - Supplemental figures, figure legends and table legends
