# Supplementary material for: Differential proteomics profiling identifies LDPs and biological functions in high-fat diet-induced fatty livers
Source: J Lipid Res. 2017 Mar 29;58(4):681–94. doi: 10.1194/jlr.M071407 (PMC5392744; doi:10.1194/jlr.M071407)
Supplement: Supplemental Data [file 10.1194_M071407_jlr.M071407-11.pdf]

Supplemental figures and figure legends

Supplemental figure S1

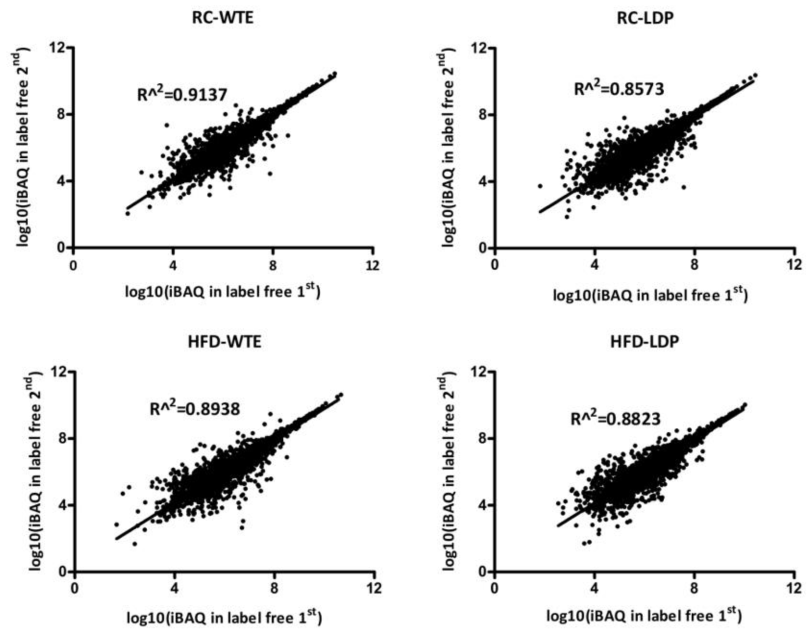

Supplemental figure S1. Technological repeatability of label-free quantification. Correlation curve of iBAQ value that was calculated and transformed to base-10 logarithms for proteins identified in RC_WTE&LDP and HFD_WTE&LDP.

Supplemental figure S2

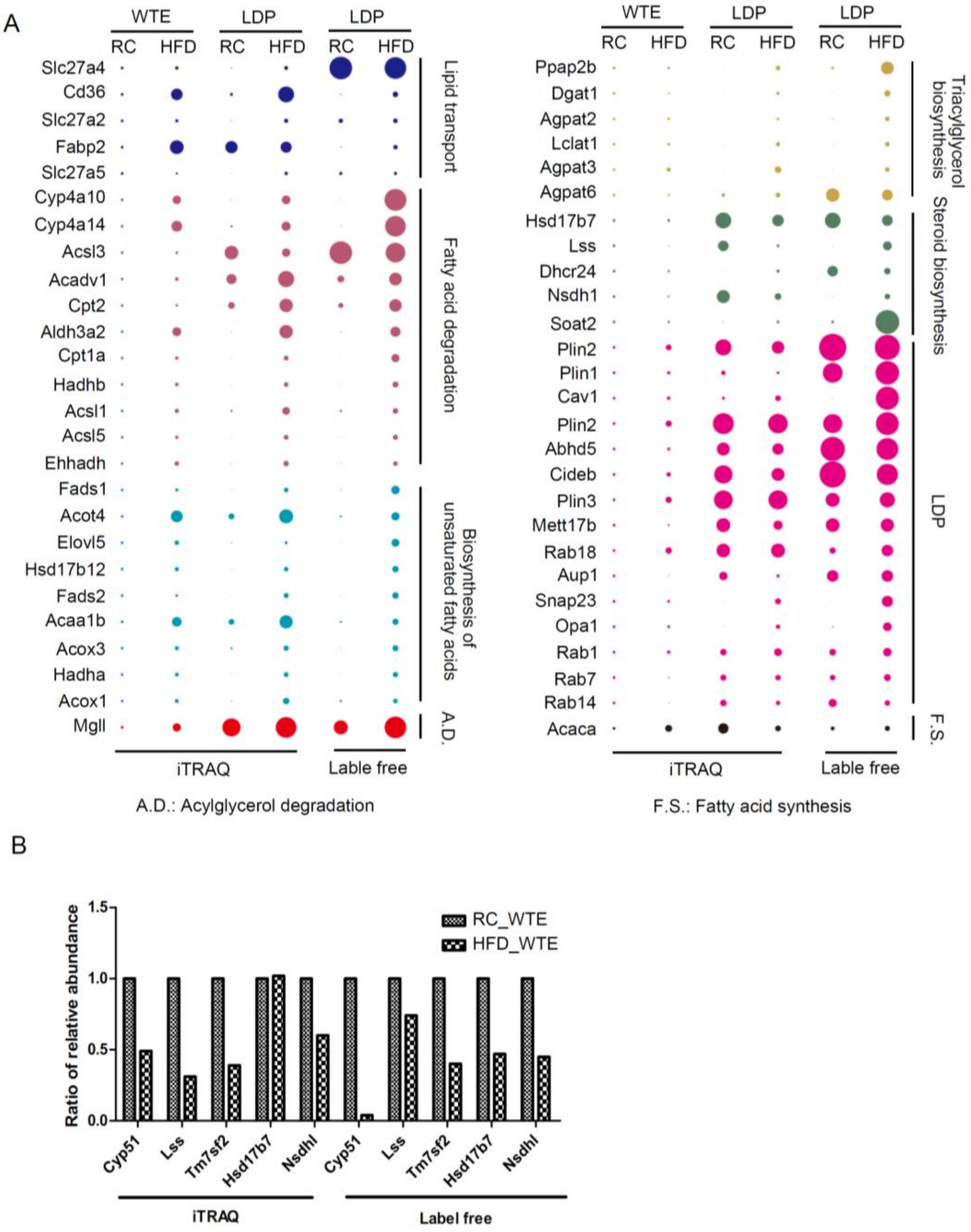

Supplemental figure S2. Quantification of 8 major biological processes by using label-free quantification approach. (A) Up-regulation of proteins in HFD WTE and LD on acylglycerol degradation, biosynthesis of unsaturated fatty acids, fatty acid degradation, LDP, lipid transport, steroid biosynthesis, and triacylglycerol biosynthesis, in comparison to those in RC WTE and LD. (B). Comparison of 5 enzymes in the consecutive enzymatic reactions in steroid biosynthesis among iTRAQ and label free methods.

Supplemental figure S3

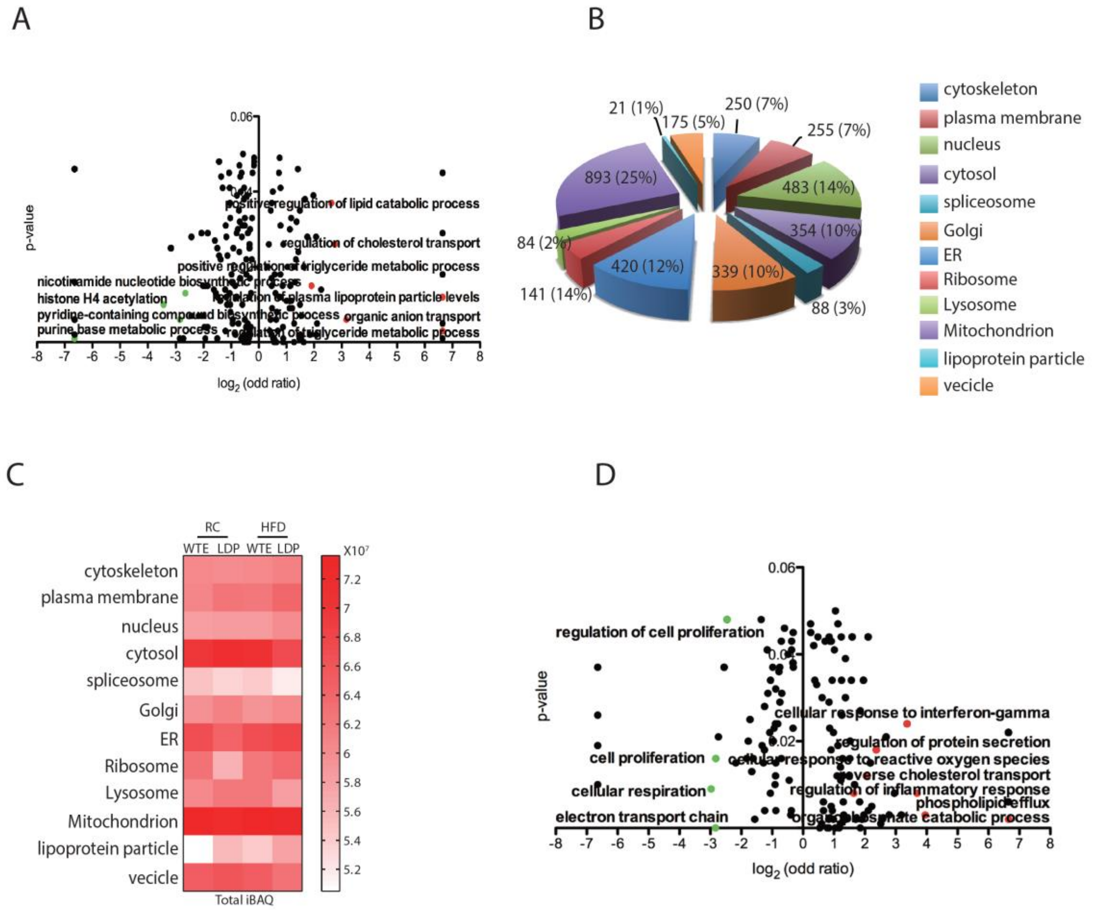

Supplemental figure S3. (A) Volcano plot of biological processes of LD sub-proteome compared to liver global proteome, in physiology condition. (B-C) GO analysis and comparison of liver global proteome and LD sub-proteome. (B) Frequency of GOCC terms in LD sub-proteome of RC mice liver. (C) Comparison of absolute abundances of GOCC terms in liver global proteome and LD sub-proteome from fatty liver and RC fed mouse. (D) Volcano plot of biological processes of differential proteome in mouse fatty liver.

Supplemental figure S4

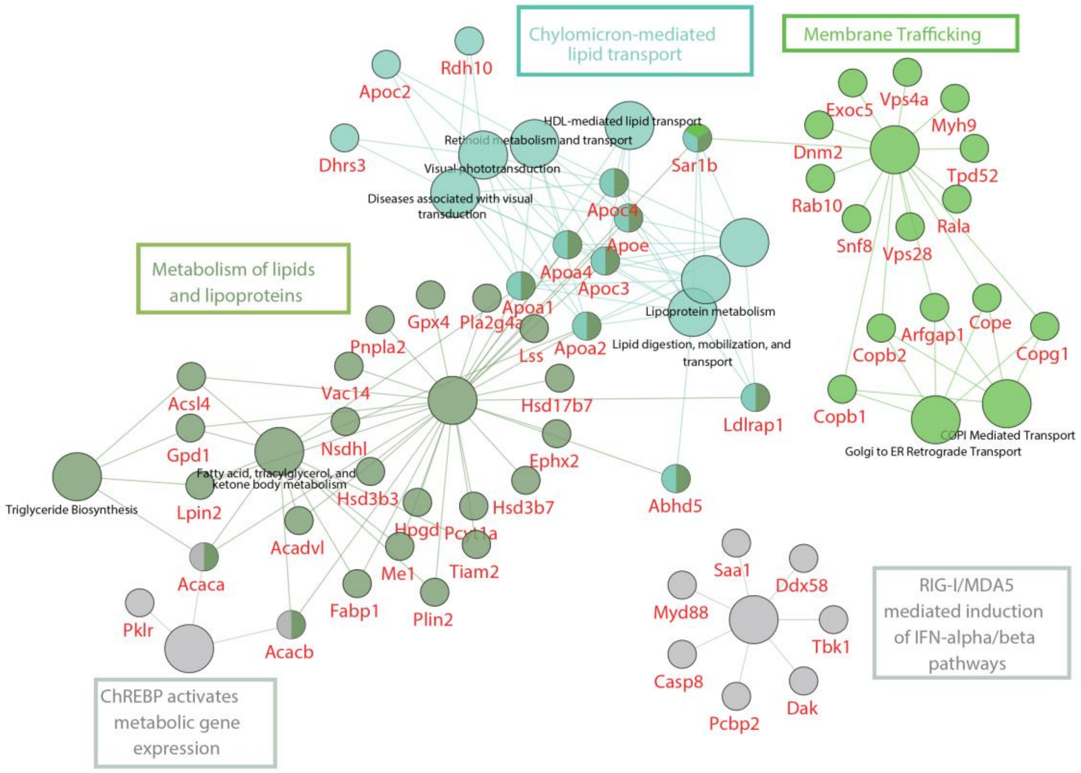

Supplemental figure S4. Bioinformatics analysis revealed top LD enriched proteins were dominantly included in 5 major biological processes.

Supplemental figure S5

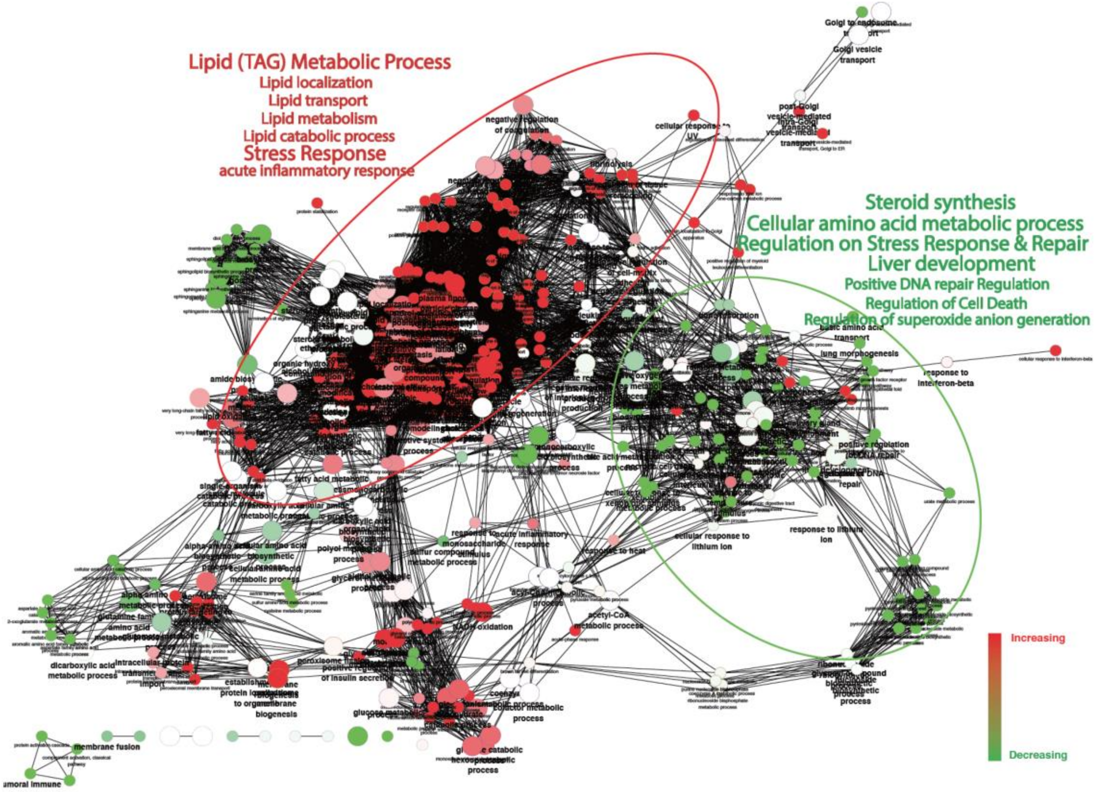

Supplemental figure S5. Significant enriched GO biological process term annotation of differentially expressed proteins in fatty liver group compared with RC mice. The color from green to red represents down-regulation and up-regulation respectively; size of nudes represents significance of GO enrichment.

Supplemental figure S6

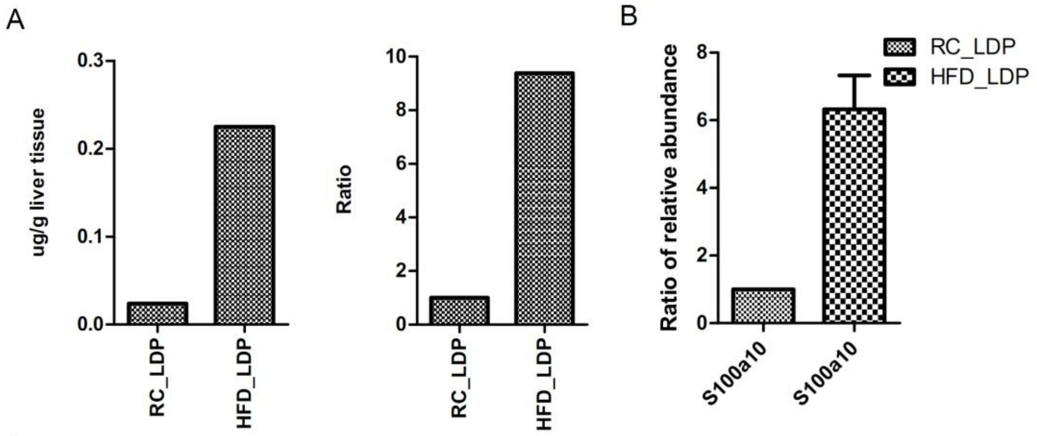

C

Raw data for a precursor of S100a10 analysed in MRM runs							
				sh-control			
Precursor	CE	charge	M/Z	Transition	Area	RT	sh-S100a10
PSQMEHAMETMMLTFHR	28.6	3	692.9762	459.2694	187952	68.07	Not available
PSQMEHAMETMMLTFHR	28.6	3	692.9762	693.31537	383515	68.17	Not available
PSQMEHAMETMMLTFHR	28.6	3	692.9762	426.1995	156812	68.17	Not available

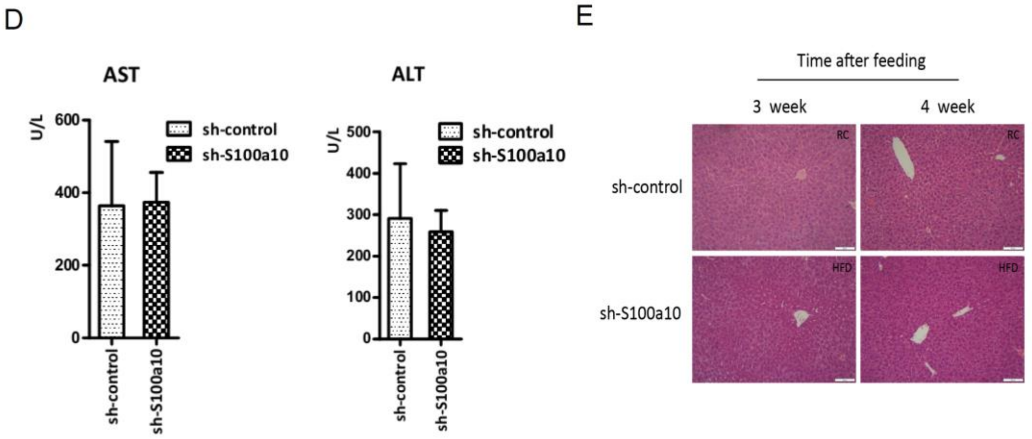

Supplemental figure S6. Knockdown efficiency of hepatic S100A10 was evaluated by MRM-based proteomics approach. (A) Comparison of the LDP amount that was isolated from RC and HFD liver tissue. The LDP amount from RC fed mice was set as 1. (B) Ratio of S100a10 relative abundance through label-free approach. (C) Raw data for a precursor of

S100a10 analyzed MRM runs. (D) Serum AST and ALT of mouse after one injection of S100A10-shRNA recombinant adenovirus for two weeks (n=3). Data are mean ± SD. Two tailed student's t test was performed (*, $p < 0.05$; **, $p<0.001$).(E) H&E staining of mouse liver under HFD administration for 3 and 4 weeks (Scale bars, 50μm.).

Supplemental figure S7

A

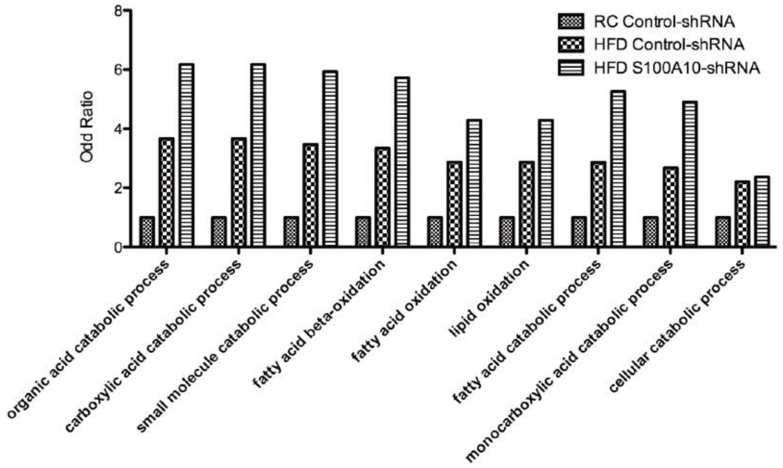

Supplemental figure S7. Regulation of lipid metabolism processes of HFD fed mice administrated with control-shRNA and S100A10-shRNA, in comparison to regular chaw mice administrated with control-shRNA.

Supplemental table legends

Supplemental Table S1. Evaluation the purity of isolated LD in intracellular organelles by using parallel reaction monitoring (PRM).

Supplemental Table S2. Proteins identified and quantified in whole liver protein and LDP both in regular chaw (RC) and fatty livers.

Supplemental Table S3. The enrichment factors of 101 “core” LDPs in RC group.

Supplemental Table S4. Proteins identified in both iTRAQ and label-free approach.

Supplemental Table S5. Relative abundance of proteins in LDP and other 6 lipid metabolic processes.

Supplemental Table S6. Over- and under-represented biological processes of LDPs.

Supplemental Table S7. Enrichment factors of proteins identified in LDP of RC and HFD groups.

Supplemental Table S8. Label free quantification for S100a10 in LDP of RC and HFD groups.

Supplemental Table S9. Proteomic analysis of proteins identified in S100a10 knockdown and control mice liver after 4 weeks of HFD feeding.

Supplemental Table S10. Biological processes analysis of S100A10 interactome
